# Supplementary material for: FERN – a Java framework for stochastic simulation and evaluation of reaction networks
Source: BMC Bioinformatics. 2008 Aug 29;9:356. doi: 10.1186/1471-2105-9-356 (PMC2553347; doi:10.1186/1471-2105-9-356)
Supplement: Additional file 1 — FERN distribution, Version 1.3. This archive contains the FERN source code and binaries as well as documentation and example models in FernML and SBML. [file 1471-2105-9-356-S1.zip › fern/doc/javadoc/fern/simulation/algorithm/IndexedPriorityQueue.html]

IndexedPriorityQueue


---


|  |  |  |
| --- | --- | --- |
| **Overview** \| **Package** \| **Class** \| **Use** \| **Tree** \| **Deprecated** \| **Index** \| **Help** | |  |
| **PREV CLASS**   **NEXT CLASS** | **FRAMES**    **NO FRAMES**     **All Classes** |  |
| SUMMARY: NESTED \| FIELD \| CONSTR \| METHOD | DETAIL: FIELD \| CONSTR \| METHOD |  |


---


## fern.simulation.algorithm Class IndexedPriorityQueue

```
java.lang.Object
  fern.simulation.algorithm.IndexedPriorityQueue
```

---

``` public class IndexedPriorityQueue extends Object ```

Implements an indexed priority queue. It is a data structure to manage the absolute taus
of the Gibson-Bruck algorithm in an efficient way. It is composed of a binary heap (the
taus are the keys) and an index for fast access to a node of the heap. The fast access
is necessary to update unused taus in the queue.

For reference see M.A.Gibson and J.Bruck, Efficient Exact Stochastic Simulation of Chemical
Systems with Many Species and Many Channels, J.Phys.Chem.A., Vol 104, no 9, 2000

**Author:**
:   Florian Erhard

**See Also:**: `GibsonBruckSimulator`

---

| **Field Summary** | |
| --- | --- |
| `static Random` | `RND` |


**Constructor Summary**

`IndexedPriorityQueue(double[] t)`
  
          Creates a `IndexedPriorityQueue` from the given taus


| **Method Summary** | |
| --- | --- |
| `double` | `getKey(int i)`             Gets the tau of a reaction by lookup in the index |
| `int` | `getMin()`             Gets the index of the minimal tau in the queue. |
| `double` | `getMinKey()`             Gets the minimal tau in the queue. |
| `int` | `size()`             Gets the number of nodes in the binary heap. |
| `String` | `toString()` |
| `void` | `update(int i, double value)`             Updates the tau of a reaction to a given value |

**Methods inherited from class java.lang.Object**

`clone, equals, finalize, getClass, hashCode, notify, notifyAll, wait, wait, wait`

**Field Detail**

### RND

```
public static final Random RND
```


**Constructor Detail**

### IndexedPriorityQueue

```
public IndexedPriorityQueue(double[] t)
```

:   Creates a `IndexedPriorityQueue` from the given taus

    **Parameters:**: `t` - taus


**Method Detail**

### toString

```
public String toString()
```

:   **Overrides:**: `toString` in class `Object`

---


### size

```
public int size()
```

:   Gets the number of nodes in the binary heap.

    :   **Returns:**: number of nodes in the binary heap.

---


### getMin

```
public int getMin()
```

:   Gets the index of the minimal tau in the queue.

    :   **Returns:**: index of the minimal tau

---


### getMinKey

```
public double getMinKey()
```

:   Gets the minimal tau in the queue.

    :   **Returns:**: minimal tau

---


### update

```
public void update(int i,
                   double value)
```

:   Updates the tau of a reaction to a given value

    :   **Parameters:**: `i` - index of the reaction: `value` - new tau

---


### getKey

```
public double getKey(int i)
```

:   Gets the tau of a reaction by lookup in the index

    :   **Parameters:**: `i` - index of the reaction **Returns:**: tau of the reaction


---


|  |  |  |
| --- | --- | --- |
| **Overview** \| **Package** \| **Class** \| **Use** \| **Tree** \| **Deprecated** \| **Index** \| **Help** | |  |
| **PREV CLASS**   **NEXT CLASS** | **FRAMES**    **NO FRAMES**     **All Classes** |  |
| SUMMARY: NESTED \| FIELD \| CONSTR \| METHOD | DETAIL: FIELD \| CONSTR \| METHOD |  |


---
